# Endometriosis: Future Biological Perspectives for Diagnosis and Treatment

**DOI:** 10.3390/ijms252212242

**Published:** 2024-11-14

**Authors:** Mary Garvey

**Affiliations:** Department of Life Science, Atlantic Technological University, Ash Lane, F91 YW50 Sligo, Ireland; mary.garvey@atu.ie; Tel.: +071-9305-529

**Keywords:** endometriosis, morbidity, inflammation, dysbiosis, infertility, ATMPs, microfluidics

## Abstract

Endometriosis is an oestrogen-dependent inflammatory disease affecting menstruating women, with varying levels of severity. Oestrogen dysregulation is responsible for chronic inflammation, angiogenesis, endometrial lesion development, progression, and infertility during menarche in afflicted women. The inflammatory mediators associated with this chronic painful disease have been established, with research also indicating the relationship between dysbiosis and disease manifestation. Endometriosis is also present with several painful comorbidities, including endometrial cancer, cardiovascular disease, and autoimmunity. The lack of specific and sensitive non-invasive diagnostic procedures, coupled with poor response to current therapeutic approaches, means that treatment needs remain unmet. Surgical procedures are performed to remove endometriosis ectopic lesions, for which the recurrence rate of disease is up to 50%, with certain patients exhibiting no alleviation of symptoms. This review aims to outline the aetiology of endometriosis, detailing novel diagnostic approaches and potential therapeutic approaches, namely advanced therapeutic medical products (ATMPs), including stem cell therapy and clustered regularly interspaced short palindromic repeats (CRISPR) gene editing. This timely review also provides novel insights into the important recent modalities which may be applied for the diagnosis and therapeutic response of endometriosis, including biomarkers, microfluidic platforms, and organoid systems. Undoubtedly, reliable, reproducible, sensitive, and specific models of endometriosis in humans are urgently needed to investigate and detail the aetiology of this debilitating disease.

## 1. Introduction

Endometriosis is a progressive inflammatory disease, dependent on the hormone oestrogen, in which endometrial tissue migrates and implants ectopically in locations outside of the uterine cavity. This endometrial tissue is commonly present in the uterine muscular layer, the ovaries, and areas outside of the reproductive tract, including the peritoneum, urinary system, and the gastrointestinal tract (GIT) [1]. Migratory endometrial tissue produces its own oestrogen and interacts with the body’s endocrine, musculoskeletal, vascular, reproductive, and nervous system, resulting in the symptoms of endometriosis [2]. The World Health Organisation (WHO) describes endometriosis as a complex disease affecting women of reproductive age, with a global prevalence of 10% [3]. The precise prevalence of endometriosis, however, is believed to be closer to 15% [4]. Endometriosis can be present asymptomatically, with minimal or no symptoms, which impacts disease diagnosis and surveillance. Endometriosis occurs between menarche and menopause; however, it can be present in post-menopausal women [4]. Studies report that endometriosis is present in ca. 70% of women, manifesting with dysmenorrhea (painful menstruation) and dyspareunia (pain during and/or after sexual intercourse), with adolescent patients exhibiting more severe symptoms [5]. Endometriosis is also evident in ca. 80% of women manifesting with pelvic pain and ca. 50% of infertile women [6]. When present clinically or symptomatically, symptoms are evident, with varying levels of severity. Clinical endometriosis is associated with pelvic pain, painful ovulation, lower back pain, menorrhagia (heavy menstruation), dyschezia (painful defecation), dysuria, dysmenorrhea, and GIT issues [4].

The type and severity of endometriosis present is categorised as stage I to IV (Table 1), with symptoms dependent on the extent of the disease and the organs affected. It must be noted, however, that the severity of pain symptoms does not always correspond with the stage of disease present, where stage III or IV, for example, may be less symptomatic than stage I. Undoubtedly however, this chronic condition negatively impacts a patient’s physical, sexual, psychologic, and social health, as the severity of symptoms, including pain, is unpredictable and often unmanageable in patients. Importantly, studies show that endometriosis patients have significantly higher rates of comorbidities, including ovarian and endometrial cancer [7], cardiovascular disease, autoimmune disease, and mental illness than do non-endometriosis patients [8]. Additionally, patients have a higher risk of miscarriage and ectopic pregnancies than their unaffected counterparts [9]. Studies show that endometriosis patients have an increased risk of placenta praevia, unexplained antepartum haemorrhage, postpartum haemorrhage, and preterm births [10]. Current theories suggest that endometriosis is multifactorial in its aetiology, with genetic, epigenetic, immunological, hormonal, and possibly GIT and endometrium microbiota, involvement [11]. Oestrogen dominance is a feature of endometriosis contributing to immune dysregulation [12]. The human microbiota is composed of hundreds of microbial species, primarily residing in the GIT [13], which are involved in maintaining normal peritoneal integrity and ectopic cell clearance [14]. The role of the microbiota and an imbalance in the species present, termed dysbiosis, is associated with many chronic morbidities [15]. There is a significant delay (4 to 11 years) in the diagnosis of endometriosis from onset of symptoms, with confirmation via laparoscopy and pathological examination of tissue deemed the gold standard approach. The lack of biomarkers, specific and sensitive non-invasive methods, and a detailed understanding of endometriosis aetiology contributes to a lack of diagnosis and to misdiagnosis [16]. Symptoms are variable and heterogenic amongst patients, complicating disease management. Often, cases of endometriosis remain unconfirmed, particularly in resource-limited developing countries, meaning that the true burden of the disease remains unknown [17]. Additionally, endometriosis is often misdiagnosed as appendicitis, irritable bowel syndrome (IBS), pelvic inflammatory disease, and food intolerance [2]. This chronic, progressive, and debilitating disease results in patients reporting a lower health-related quality of life (HRQoL) and increased economic burdens [18]. There is an urgent need for improved diagnostic and treatment protocols for this difficult to treat disease, as current medical approaches remain deficient. This article provides insights into the aetiology of endometriosis and the limitations of current diagnostic and treatment options and reviews recent advances that may aid in disease management.

## 2. Pathophysiology of Endometriosis

The revised American Society for Reproductive Medicine (rASRM) system is the most recognised system for classifying endometriosis pain according to severity [16]. Currently, endometriosis is divided into three categories according to physiopathology, localization, and ectopic endometrial lesions containing endometrial glands and stroma, i.e., superficial peritoneal, ovarian, and deep infiltrating lesions, classified into stages I–IV [19]. Peritoneal endometriosis is the most common category, affecting ca. 44% of women, followed by ovarian and deep infiltrating endometriosis, which is the most severe form (Table 2) [2]. The exact aetiology of the disease remains undetermined; however, theories suggest that migratory endometrial tissue is a result of retrograde menstruation, causing endometrial tissue to pass through the fallopian tubes during menstruation due to uterine contractions and to accumulate in areas outside of the uterine location; this is known as Sampson’s assumption [9]. Post translocation, the ectopic tissue undergoes neuro-angiogenesis and induces a local inflammatory response, fibrosis/scarring, and pain in patients [20]. Retrograde menstruation does not provide sufficient causation for deep infiltrating endometriosis, however, or for the presence of lesions in visceral tissues [21]. Furthermore, retrograde menstruation is present in ca. 90% of menstruating women, with most not developing endometriosis [22]. A combination of factors, including endometrial stem cell implantation, coelomic metaplasia (Gruenwald’s theory), and immune system dysregulation, are also potential contributing factors [23]. Gruenwald’s theory suggests the ability of parietal peritoneum epithelium to differentiate into endometrial tissue, when influenced by cytokines and growth factors from the endometrial stroma [21], and offers an explanation for the presence of the disease in women who do not have a uterus or endometria or patients with Mayer–Rokitansky–Küster–Hauser syndrome [24]. Additional theories include the embryogenetic theory that oestrogen causes endometrial tissue to develop from embryonic remnants, producing endometriosis lesions, and the stem cell theory, which suggests that endometrial and hematopoietic stem cells differentiate into endometrial cells and form endometriotic lesions [25]. As endometriosis is a hereditary disease, genetic mutations are associated with the development of endometriosis, including an increase in mutant allele frequencies (MAF) [26]. Endometriosis shares many immune biological traits present in certain cancers, including accelerated somatic mutations in the endometrial epithelial cells [12]. Indeed, research suggests that endometriosis may be an initial step in the development of ovarian cancer [27]. Endometriosis is an oestrogen-responsive disease, with 17β-oestradiol prompting the proliferation of the growth of ectopic endometriosis tissue via receptor binding of oestrogen receptor α (ERα) and estrogenic receptor β (ERβ) [28]. Studies show an increased number of 17β oestradiol receptors in ectopic endometrium tissues compared to that in eutopic and non-endometriosis women [29]. The administration of combined oestrogen and progesterone contraceptives are a front-line approach to the treatment of this oestrogen-dominant condition [12]. The therapeutic application of gonadotropin-releasing hormone (GnRH) agonists to inhibit oestrogen production is also applied in the treatment of endometriosis [30]. Unfortunately, such therapies are often unsuccessful in eliminating symptoms and preventing lesion growth [31]. In such cases, surgical removal of endometriosis lesions via laparoscopy is performed to provide symptom relief and restore fertility; however, ca. 50% of patients relapse, displaying lesion regrowth [32].

### 2.1. Immune Dysregulation

Immune cells play a vital role in regulating and removing endometrial tissue in menstruating women; alterations in the functioning of the immune system contribute to the pathogenesis of endometriosis [35]. Recent studies suggest the contribution of immune dysregulation and pro-inflammatory mediators in the aetiology of endometriosis in women having an altered endometrium immune environment [21] (Table 3). Cells of the immune system which promote the proliferation of endometrial lesions include macrophages, neutrophils, natural killer (NK cells), activated mast cells, dendritic cells, and T cells [20]. The progression of endometriosis is impacted by specific anti-inflammatory cytokines, including interleukins (IL) IL-1 beta, IL-5, IL-6, IL-7, IL-12, IL-4, and IL-10, which contribute to the persistence, expansion, infiltration, differentiation, angiogenesis, and immune evasion of endometriotic lesions [36]. Importantly, endometrial cells of the menstruation phase secrete a higher amount of IL-6, which displays pro-inflammatory action and stimulates endometrial mesenchymal stem cells (eMSC) [37]. T cells are categorised as cytotoxic T lymphocytes (CTLs), T helper (Th) cells, and regulatory T cells (Tregs), with Th cells further categorised as Th1, Th2, Th17, and Treg subsets, having a distinct cytokine profile.

Macrophages exert a phagocytic action on rouge menstrual tissue in healthy women; in endometriosis patients, however, they display a reduced action, ultimately promoting lesion formation, angiogenesis, and fibrogenesis, causing chronic inflammation via cytokine excretion [38]. Pro-inflammatory cytokines excreted by macrophages in the peritoneal fluid induce inflammation, the development and progression of endometriosis invasion, and lesion formation [39]. The research of Zou et al. (2021) identified two new macrophage subtypes in the peritoneal fluid of endometriosis patients, namely the TCR^+^ macrophages and proliferating macrophages [35]. When ectopic lesions adhere to the peritoneum, cytokines, growth factors and vascular endothelial growth factor (VEGF), tumour necrosis factor α (TNF α), and IL-8 encourage angiogenesis and lesion proliferation [40]. Macrophage action in endometriosis also results in neuroinflammation and progesterone resistance [31]. Progesterone resistance in the endometrial tissues allows for the survival and growth of the migratory cells ectopically and diminishes the action of progestin-based pharmacological therapy approaches [34]. Leukocytes, including cytotoxic T lymphocytes and NK, also contribute to the pathophysiology of endometriosis; the reduced action of the T cells allows for the immune avoidance of the endometriotic tissue, with endometriosis cells triggering the apoptosis of T cells via receptor binding [31]. Studies show elevated levels of NK and T cells present in endometrial tissue in which their activity is decreased and their phagocytic role in ectopic cell clearance is not performed [35]. T helper 17 cells (Th17 cells) secrete IL-17, a pro-inflammatory cytokine which stimulates the expression of angiogenic activity, promoting angiogenesis in the ectopic endometrial lesions [41]. Research has identified increased levels of Th 17 cells and corresponding cytokine IL-17 in peritoneal endometrioses in which severe disease and infertility is evident [42].

The peritoneal fluid also contains increased numbers of proinflammatory CD8 T cells compared to that in the peripheral blood [43]. Degranulated mast cells are also abundant in ectopic lesions, releasing pro-inflammatory mediators including histamine, TNF- α, and IL-6, sensitising local sensory nerves, resulting in hyperalgesia [44]. IL-6 is very active in cardiac disease, autoimmune diseases, cancer, and the cytokine storm, with astrocytes, neurons, and microglia containing IL-6 receptors [45]. IL-17 is also excreted by activated CD8^+^ T cells, γδ T cells, NK cells, neutrophils, and mast cells in the peritoneal cavity [46]. Oestrogen activates pro- and anti-inflammatory immune pathways for all immune cells, e.g., macrophages, T cells with ERα and ERβ receptors, and some with progesterone receptors, altering inflammatory action in the endometrial tissue [47]. Studies show an increase in the presence of sensory nerve fibres in endometriosis patients with a decrease in sympathetic fibres present compared to that for non-endometriosis persons, leading to chronic neurogenic pain signalling [48]. ERs are also present on nerve fibres, and endometriotic lesions are innervated, causing them to actively recruit macrophages to the ectopic endometrial tissue, inducing neural hypertrophy and increased neural density in the endometriotic lesion, leading to inflammatory, nociceptive, and neuropathic pain [49].

**Table 3:** Inflammatory mediators associated with endometriosis aetiology.

Inflammatory Mediators	Endometrial Effect	Clinical Manifestations
Interleukins (ILs)—cytokines	Produced by macrophages, IL-6 impairs the cytotoxic function of NK cells [50], with an innate and adaptive pro-inflammatory effect [25]. IL-8 recruits neutrophils which produce IL 17A, VEGF, and cyst formation [51]; IL-19 and IL-22 are immunosuppressive and anti-inflammatory; IL-19 promotes Th2 cells, increasing the expression of IL-10 [25].	Upregulation leads to cytokine imbalance and pro-inflammatory action, inducing pain [51]. Downregulated anti-inflammatory ILs causes endometrioma to evade immunosurveillance.
Tumour necrosis factor (TNF)-α,	Activates nuclear factor κB (NF-κB) and hypoxia inducible factor (HIF)-1α signalling pathways, increasing cyclooxygenase (COX)-2 expression in endometriosis [52].	Autocrine and/or paracrine cytokines regulating local immune and inflammatory responses; expression of COX-2 in peritoneal macrophages influences the severity of endometriosis, dysmenorrhea, and infertility [52]; central hyperexcitability from TNF-α [53].
Prostaglandins PGE_2_ and PGF_2α_	Modulates the balance ratio of Th1/Th2, regulates chemokine secretions, and inhibits lymphocyte alloreactivity.	Pro-inflammatory action leading to chronic pelvic pain [54].
Leukocytes—T cells 60% CD8+ T (CD8 T) Regulatory T (Treg) cells and T helper cells NK cells	Secrete pro-inflammatory cytokines (IFN-γ and TNF-α). Decrease in T reg population leads to increased systemic and local inflammation within ectopic and eutopic endometrium [55]. Kill the antigen-positive cells with cytolytic molecules perforins and granzymes [42]; NK cell cytotoxicity is diminished in patients [56].	Pelvic pain, fertility issues, miscarriage; CD8 T is associated with autoimmune disease [43]. Chronic inflammation, strong immunosuppressive activity [55]; dysmenorrhea, dyspareunia, non-menstrual pelvic pain, rectorrhagia, dyschezia [56].
Neutrophils	Human neutrophil peptides (HNP 1–3) and neutrophil-activating peptide (ENA)-78 secrete cytokines CXCL10, and IL-8 secretes VEGF.	Severity of dysmenorrhea and chronic pelvic pain [31]; peritoneal immuno-inflammation, development of endometriosis; endometriotic stromal cells promoting endometriosis progression via lymphangiogenesis [57].
Leptin	Hormone with immunoregulatory, proinflammatory, and angiogenic effects [58]; cytokine promoting CD4+ T helper I cell proliferation, macrophage phagocytosis, secretion of inflammatory cytokines [59], production of prostaglandins.	Stimulates activation of peritoneal macrophages and increases pro-inflammatory activity and pain [59].
Oestrogen (17β-oestradiol)	Binding to oestrogen receptor α and β, inducing proliferation [28], increases neutrophils concentration and expression of pro-inflammatory cytokines, induces the expression of semaphorins in uterine tissue.	ERβ expression is significantly higher in ectopic endometrium [28]; establishment and progression of endometriosis.
Neuropeptides	Substance P (SP), calcitonin gene-related peptide (CGRP) present in endometrial lesions having proinflammatory activity. Brain-derived nerve growth factor (BDNF) and neurotrophin 3 (NT3).	Activation of sensory afferent nerves and chronic state of neurogenic inflammation; neurogenesis and peripheral sensitization, hyperalgesia [44].

### 2.2. Dysbiosis and Endometriosis

The impact of dysbiosis on human health has long been recognised. An imbalance in the commensal microbial species residing in and on the body contributes to many disease states, including autoimmunity, cancer, and mental health issues [15]. More recently, the role of the microbiota in the establishment and proliferation of endometriosis has gained interest. Furthermore, endometriosis patients are at increased risk of developing mental health illnesses and autoimmune disease, suggesting a causality crossover. The rate of major depressive disorder in endometriosis patients was determined to range from 1.36% to 55.3%, with the rate of anxiety disorder ranging from 3.7% to 29.7% in endometriosis patients [60]. The bacterial microbiota of the GIT is dominated by the phyla *Firmicutes*, *Actinobacteria*, and *Bacteroidetes*, which are essential in regulating host immunity, GIT endocrine function, neurological signalling, food digestion, xenobiotic detoxification, and elimination, producing essential biological compounds for the human host [13].

A healthy GIT typically contains a lower abundance of *Verrucomicrobia* and *Proteobacteria.* Intestinal permeability or leaky gut allows microbes, toxins, and other particles to enter the bloodstream, triggering an immune response and potentially, chronic inflammation, which may lead to the progression of endometriosis [61]. Studies have shown that the GIT microbiota are involved in the regulation of oestrogen [62]. Research demonstrates that gut dysbiosis leads to increased levels of systemic oestrogen that may be associated with the hyper-estrogenic environment and the progression of endometriosis [63]. A dysbiosis of the estrobolome, the microbiome of the microbial species involved in oestrogen metabolism, contributes to the presence of excess oestrogen in the body and may impact endometriosis formation and progression [64]. Mice models have identified GIT dysbiosis in endometriosis animals, with an increased presence of *Firmicutes* in the endometriosis group compared to that in the controls, which exhibited an increased presence of *Bacteroidetes* [65]. Species such as *Clostridia* and *Ruminococcacaeae* spp. produce active oestrogens through deconjugation and influence oestrogen-dependent diseases via beta-glucuronidase activity, reducing the elimination of oestrogen and increasing blood circulating levels, which may influence endometriosis lesion formation and progression [66]. The ectopic endometrial environment and the peritoneal cavity in endometriosis patients has been determined to be non-sterile [67]. The pathological effect of dysbiosis in endometriosis may be via inflammatory pathways and immune influence, as demonstrated in endometriosis patients when compared to healthy controls in human and animal studies [68]. Studies identify a dominance of the *Actinobacteria*, *Firmicutes*, *Proteobacteria*, and *Verrucomicrobia* genera in the GIT of endometriosis patients, with a reduced presence of *Lactobacillaceae* [69,70]. Consequently, the levels of *Proteobacteria*, *Enterobacteriaceae E. coli* and *Shigella*, and *Streptococcus* were increased in endometriosis patients [69]. Studies also describe the presence of *Enterobacterale Klebsiella* in the cervix of endometriosis patients, which may relocate to the peritoneal cavity, inducing an inflammatory response [71].

Additional studies identified an excess of Gram-negative bacteria, including *Pseudomonas* and *Prevotella.* Gram-negative species, including *Pseudomonas*, *Klebsiella*, *E. coli*, and *Prevotella*, secrete a lipopolysaccharide (LPS) toxin which activates macrophages and cytokine excretion and induces an inflammatory reaction, promoting endometrial lesion formation [72]. Studies demonstrate that LPS stimulates the macrophage secretion of cytokines IL-6, TNF-α, and growth factors, including VEGF and hepatocyte growth factors, in the pelvic and peritoneal areas [54]. This aligns with the “bacterial contamination hypothesis”, put forward by Khan et al. (2018), in which the LPS toxin and Toll-like receptor 4 (TLR4) cascade may be key in the pathophysiology of endometriosis [73]. The TLR4 cascade is directly linked to the growth and progression of endometriosis lesions and subsequent pelvic inflammation. *Streptococcus* species have been detected in endometriosis patients exhibiting severe disease, with increased levels of IL-8, IL-1, and cyclooxygenase-2 (COX-2), which induce the synthesis of pro inflammatory prostaglandin E2 (PGE2) [74]. Importantly, the findings Han of et al. (2024), assessing the fungal microbiome of endometriosis patients, determined an overpopulation of *Penicillium* spp. present compared to that in the control groups [75]. The application of antibiotics to treat microbiota imbalance resulted in changes to macrophage and dendritic cells and decreased visceral pain via immune and nociceptive responses [66]. Resident microbiota species also influence the production of neurotransmitters, including γ-Aminobutyric acid (GABA), norepinephrine, dopamine, and serotonin [13], which can impact hormone levels via the hypothalamic–pituitary–adrenal (HPA) axis. Studies have shown that dysfunction of the HPA axis is present in endometriosis patients, suggesting hypocortisolaemia or low cortisol levels as a biomarker for disease [76]. The role of the microbiome and dysbiosis in endometriosis has not been fully elucidated, but research highlights the importance of this potential aetiology. The studies described provide some insight into associated imbalances as determined by animal models, which may provide possible therapeutic targets for disease treatment. Certainly, it has become evident that this is an area worthy of more focused studies using patient microbiota analysis clinically, where possible.

### 2.3. Comorbidities Associated with Endometriosis

Endometriosis has been established as a comorbidity of many autoimmune conditions, including inflammatory bowel diseases (IBDs), Crohn’s and ulcerative colitis (UC), systemic lupus erythematosus (SLE), rheumatoid arthritis (RA), and psoriasis [66]. IBD has a symptomology similar to that of bowel endometriosis, manifesting with abdominal pain, diarrhoea, weight loss, and fatigue, with a higher prevalence in females [36], with a prevalence of ca. 3.4% of IBD patients presenting with endometriosis. A dysregulation of cytokines, including IL-17, IL-23, and IL-10, is common amongst these inflammatory diseases and endometriosis [42]. Additional immune characteristics present include T cell and B cell dysfunction, decreased apoptosis, tissue damage, nociceptor stimulation, and neuropathy [54]. In cases of IBD and psoriasis, IL-17 and IFN-γ produce inflammatory mediators and degradative enzymes, leading to tissue necrosis [77]. A dysregulated TH 17 or Treg composition results in immune tolerance and promotes the chronic inflammation seen in autoimmune disease [41]. IL-6 is an active contributor to cytokine inflammatory responses via cytokine activation, neural receptor binding, B cell and IgG production, CD8+ differentiation, VEGF action, angiogenesis, and vascular permeability [78]. Studies describe the presence of endometriosis in 71% of IBD patients with dyspareunia and dyschezia as symptoms; deep infiltrating and posterior adenomyosis was also higher in IBD patients than in the controls [79]. Case studies show that the presence of endometriosis and IBD in patients leads to improper diagnosis when symptoms overlap, and poor therapeutic response is evident [80]. The research of Gan et al. (2023) determined that the risk of developing UC or Crohn’s is increased in endometriosis patients compared to that in the controls and presents evidence of shared pathological aetiology in both disease states [81].

Studies have also suggested that endometriosis is associated with SLE, RA, multiple sclerosis (MS), Addisons disease, Sjögren’s syndrome (SS), autoimmune thyroid disorder, and coeliac disease. The researchers, however, report a risk of bias in the study and could only provide high quality evidence for an association between SLE, RA, MS, and IBD [82]. Endometriosis is present within families and is considered a heritable disease, as genes, including the protein tyrosine phosphatase non-receptor type 22 gene (PTPN22), the predisposing gene for human autoimmune diseases, are also associated with endometriosis [83]. Studies determined that the genes CXCL12, PECAM1, and NGF, which are associated with immune cell macrophages and mast cells, were highly expressed in endometriosis [26].

Importantly, endometriosis is more severe in patients who have autoimmune comorbidities and may act as a significant predictor of stage IV endometriosis in patients [84]. However, these studies have certain limitations, including small sample sizes, ethnic variations, and genomic limitations; thus, large-scale studies are warranted to confirm the genetic association between endometriosis and autoimmunity.

Studies suggest endometriosis as a contributing factor in ovarian cancer, with cytokines IL-2, IL-5, IL-6, IL-8, and IL-10 detected in serum, intra-cystic fluid, and peritoneal fluid in endometriomas and ovarian cancer patient samples [85]. Some suggest endometriosis as a precursor to ovarian cancer, presenting as a precancerous lesion in clear-cell and endometrioid ovarian cancer [86]. Further studies are warranted to better establish this link between endometriosis and ovarian cancer in patients, as ovarian cancer has a 5-year survival rate of ca. 49% and is a leading cause of death in women. Indeed, endometriosis is suggested as a 50% risk factor for ovarian cancer, where patients with endometriosis have an increased prevalence of clear cell ovarian cancer and endometrioid ovarian cancer [7].

Additionally, genetic alterations, including Tp53 mutations, BCL mutations, and PTEN and ARID1A mutations, are present in both endometriosis and gynaecological cancers [87]. Studies show an increased risk of other cancers, including melanoma, non-Hodgkin’s lymphoma, and thyroid cancer, in endometriosis patients [26]. The prolonged inflammatory activity induces cytokine release, unregulated mitotic division, growth, differentiation, migration, and dysregulated apoptosis in endometriosis, which is also present in carcinogenesis [85]. Additionally, endometriosis is associated with a ca. 50% increased risk of cardiovascular disease, a ca. 20% increased risk of cerebrovascular disease, and a 56% increased risk of migraines and IBS [86]. The pathophysiology of IBS and endometriosis is similar and includes low-grade inflammation and visceral hypersensitivity [18].

## 3. Disease Management Requires Improved Diagnostic Protocols

Laparoscopy, which is an invasive and often undesirable procedure, remains the gold standard for endometriosis diagnosis. The use of instrumentation such as transvaginal ultrasound, magnetic resonance imaging (MRI), cystoscopy, computed tomography (CT), and double-contrast barium enema (DCBE) is more recently applied in the diagnosis regime, as they offer a relatively easy to apply approach aiding in clinical diagnosis [11]. These methods yield variable results, as efficacy is dependent on the radiologist’s experience and the type of endometriosis present. Such imaging methods have limited sensitivity and specificity and are therefore not able to fully replace laparoscopic procedures in the diagnosis of peritoneal lesions [33].

The first-line treatment of endometriosis relies on the use of hormone therapy, namely the contraceptive pill and non-steroidal anti-inflammatory drugs (NSAIDs) to suppress the menstrual cycle of patients and to reduce inflammation, with surgery applied to remove large endometriotic lesions, in many cases [31]. Treatment resistance remains a serious issue, with many patients failing to respond to oestrogen–progesterone combined or to single therapy (progestin) [34]. Furthermore, lesions often re-grow post-surgery (ca. 50% of patients), with multiple surgical procedures required over a patient’s reproductive lifespan [33]. More recently, the gonadotropin-releasing hormone (GnRH) agonists leuprolide acetate, goserelin, and nafarelin, or antagonists, e.g., elagolix and relugolix, are administered as second-line therapies when first-line therapy is unsuccessful in treating endometriosis [30]. GnRH agonists, however, are associated with many side effects, including vasomotor symptoms, vaginal dryness, headaches, and osteopenia, with use limited to 6 months, with antagonists affecting cholesterol levels and increasing the risk of coronary heart disease in patients [88]. GnRH therapeutics have been administered for disease treatment for over three decades. Definitive surgical interventions, i.e., bilateral salpingo-oophorectomy and hysterectomy, in women where fertility is not an issue offers symptom relief with low rates of recurrence [89]. Due to the heterogenetic nature of this condition, the clinical manifestation and responsiveness to therapy can change in individuals. Additionally, the exact evolution or progression of endometriosis lesions through the stages remains undetermined, as cases are diagnosed at stage levels, and some regression occurs in certain patient cohorts. Improved diagnostic and treatment modalities are required to improve diagnostic time and provide much-needed relief from the severe and chronic symptoms of endometriosis.

### 3.1. Diagnostic Alternatives for Endometriosis

The identification of specific biomarkers for endometriosis is urgently needed to provide non-invasive methods of diagnosis. A biomarker is a specific measurable parameter which is present in cases of disease and ideally, in disease subtypes, and absent without disease. It indicates biological activity in the presence of disease and response to treatment regimens or therapeutic intervention. The identification of suitable biomarkers may be used in conjunction with machine learning (ML) and omics studies to provide optimal point-of-care diagnostic protocols. Current research models primarily use in vivo animal models, with obvious limitations, as rodents do not menstruate and therefore, cannot mimic human reproductive diseases accurately [90].

#### 3.1.1. Immune Mediators as Biomarkers

Inflammatory pathways have a major impact on the aetiology of endometriosis; establishing inflammatory biomarkers may therefore offer improved diagnostic methodologies. The use of cytokines, plasma molecules such as CA-125, cell markers such as VEGF, and cellular molecules as biomarkers may support or replace invasive procedures [34]. Gynaecological cancers share inflammatory pathways with endometriosis, involving TNF-α, IL-1, IL-6, IL-8, and VEGF [85]. The glycoprotein CA-125 is a cancer antigen which is also elevated in endometriosis patients but is not sensitive or specific as a biomarker due to fluctuations in concentrations during menstruation [91]. CA-125 may find application in advanced endometriosis with severe pelvic adhesion, i.e., deep infiltrating endometriosis [92]. Studies have assessed the suitability of the cytokines IL-1, IL-6, IL-8, interferon-γ (IFN-γ), MCP-1, and TNF-α as biomarkers of endometriosis. The findings identified increased concentrations of Il-6, IL-8, IL-10, IL-1beta, IL-17A, TNF-α, and IFN-γ in the serum and peritoneal fluid of endometriosis patients when compared to those of healthy persons [93].

IL-6 is the most investigated cytokine in endometriosis, and its abundance may be associated with infertility in patients; its levels were significantly higher in stage I and II endometriosis, with a sensitivity of ca. 75% and a specificity of 83.3% [94]. Contradictory studies exist describing the concentration of IL-6 and other cytokines in endometriosis, with results impacted by the stage of the menstrual cycle and the concentration of progesterone present [37]. Additionally, IL-6 is a significant factor in the pathogenesis of many chronic inflammatory diseases, which is a specificity issue when diagnosing endometriosis, especially in the presence of comorbidities in patients. The application of IL6 as a diagnostic aid is therefore, greatly limited for endometriosis. VEGF A, VEGF 121, and VEGF 189 factors have been detected at elevated levels during the menstruation cycle of endometriosis patients compared to those in the control group. To act as specific biomarkers, the cut-off values for inflammatory mediators, including IFs, TNFs, and VGEF, need to be established for endometriosis as distinct from those for other inflammatory conditions. For example, studies have identified elevated levels of IL-6, IL-8, and IL-10 in cancers, with these cytokines also associated with retrograde menstruation, macrophage activation, immune evasion, and more in endometriosis patients [36]. Additional research has attempted to determine autoantibodies as potential biomarkers for endometriosis, including autoantibodies against IGF-2 mRNA-binding protein 1 (IMP1) and anti-laminin-1 antibodies [94], but cut-off values could not be determined. To successfully differentiate the endometriosis microenvironment as distinct from a tumour microenvironment or an IBD microenvironment, identifying the potential biomarkers and their corresponding cut-off values is essential. This is limited by the population sample sizes available for study, the heterogeneity of immune responses, and the overlapping function of potential biomarkers in many disease states, often present as comorbidities of endometriosis. Therefore, the application of said biomarkers is currently not feasible for specifically diagnosing endometriosis in patients and requires significant investigative studies to improve sensitivity and specificity.

#### 3.1.2. Extracellular Vesicles

Extracellular vesicles (EVs) are nano-sized vesicles which hold varying types of cellular molecules surrounded by a cell membrane and include exosomes, microvesicles, apoptotic bodies, autophagic EVs, stressed EVs, and matrix vesicles [95]. The contents of EVs include proteins, i.e., cell surface receptors, signalling proteins growth factors and cytokines, transcription factors, enzymes, extracellular matrix proteins, lipids, and nucleic acids (miRNA, mRNA, and DNA), which allow for intracellular communication, cell proliferation, development, and differentiation, and which are associated with many disease states, including heart disease, neurodegenerative diseases, and cancer [96]. The presence of membrane proteins and inflammatory mediators in EVs suggests their application as biomarkers of disease and possible therapeutic targets. Studies have assessed the presence of EVs in the lesions and stroma of endometriosis patients, where they influence cell proliferation, immune escape, angiogenesis, and lesion invasion [97]. The studies of Abudula et al. (2022) demonstrate that EVs derived from endometrial stromal cells induced normal endometrial stromal cell migration, angiogenesis, and upregulated pro-inflammatory cytokines in ovarian cells [98]. At present, there are limited data on the sensitivity and specificity of EVs as diagnostic markers of endometriosis. The study of Li et al. (2020) determined a sensitivity of ca. 81% and a specificity of ca. 71% for EV VEGF as a biomarker of endometriosis [57], with the research of Zhang et al. (2020) assessing EV microRNAs as biomarkers of endometriosis, offering unconclusive results due to limited sample sizes [99]. An excellent review of EVs in the diagnosis of endometriosis is provided elsewhere [97].

#### 3.1.3. Dysbiosis Biomarkers

As studies suggest a correlation between intestinal and local dysbiosis in endometriosis patients, establishing the presence of an imbalance in microbial species may aid in diagnostics. Identification of key species in ectopic and eutopic endometriosis during each stage of the menstrual cycle at each stage of the disease may indicate disease progression. Microbial species associated with EVs in the peritoneal fluid may also offer insight into the disease. The application of the next-generation sequencing (NGS) technique and/or bacterial cultivation allows for microbial identification. The studies of Pérez-Prieto et al. (2024), using metagenome analysis to assess the impact of the gut microbiome on the pathogenesis of endometriosis, did not find a confirmatory relationship to suggest causality or genetic biomarkers [100]. The studies of Chang et al. (2022) suggested potential microbial biomarkers for the different stages of endometriosis as follows: stage I and II, *L. jensenii*, *Corynbacteriales*, *Porphyromonadaceae*, and *Ruminococcaceae*, and for stage III–IV, *Bifidobacterium breve* and *Streptococcaceae* members (e.g., *Streptococcus agalactiae*) [101]. The studies of Chadchan et al. (2023) determined increased levels of the microbiota-derived metabolites 2-aminohepatonic acid, N-Acetyl Aspartic acid; Maltose, lactic acid, and quinic acid in faecal samples of mice with endometriosis, suggesting microbial metabolites as possible biomarkers. The serum of endometriosis patients contained elevated levels of citrate, lactate, 3-hydroxybutyrate, alanine, leucine, valine, threonine, lysine, glycerophosphatidylcholine, succinic acid, and 2-hydroxybutyrate in another study, with decreased levels of lipids, glucose, isoleucine, and arginine [102]. Currently, research reports regarding the establishment of microbial biomarkers are contradictory, and these results are greatly impacted by sample size, the heterogenicity of the disease, varying stages of disease, and the stage of the menstrual cycle. Studies have established the role of microbiota and dysbiosis in many disease states, including polycystic ovary syndrome (PCOS), infertility, IBD, psoriasis, RA, mental illnesses, and cancer [13,15]; its role in endometriosis, therefore, cannot be ignored. The interesting hypothesis of Khan et al. (2018), termed the “bacterial contamination hypothesis”, involving the LPS toxin activation of TLR4 [73], coupled with the presence of *E. coli* (and other Gram-negative species) in the endometrial microenvironment, may suggest the LPS toxin as a possible biomarker in some cases of endometriosis and suggest a therapeutic target for cases testing positive for this toxin. Certainly, additional studies are warranted to determine the presence of microbial biomarkers in peritoneal fluid, serum, and the endometriosis lesions in patients suffering the different stages of endometriosis in order to determine the optimal diagnostic protocol and the feasibility of application.

### 3.2. Microfluidics and Organoids as Diagnostic Aids

As stated, advancements in the diagnostics of endometriosis are limited by sample sizes and patient heterogenicity of the disease. Microfluidics may offer an in vitro alternative, allowing for ethical investigative studies into disease aetiology (Table 4). Microfluidics or organ-on-chip technology are in vitro assays utilising microfluidic and tissue engineering disciplines to synthesise or replicate an in vivo system closely resembling the organ or system of study [103]. Coupled with omics technology and machine learning, such assays provide enhanced sensitivity and specificity for the analysis of disease pathologies and treatment. Additionally, microfluidic systems can be analysed using an array of analytical techniques such as fluorescence microscopy, mass spectrometry, enzyme-linked immunosorbent assays (ELISA), and high-performance liquid chromatography (HPLC), amongst others [104]. Three different microfluidic models, namely, the Solo-MFP, Duet-MFP, and Quintet-MFP, have been developed to represent the tissue environment for the 28-day duration of the human menstrual cycle using mice tissue. The Solo-MFP and Duet-MFP systems were designed for single-tissue cultures, and the Quintet-MFP was designed for multiple-tissue cultures. An excellent review of the varying types of microfluidics developed to model the female reproductive tract is provided by Deng et al. (2024) [105]. Xiao et al. (2017) have developed a microfluidic system modelling the female reproductive tract (ovary, fallopian tubes, uterus, and cervix) using human cells, including additional organs and the hormone release of the menstrual cycle, termed EVATAR [106]. This setup is also oestrogen hormone-responsive due to the presence of receptors. With the addition of immune cells, namely macrophages, NK cells, etc., such a system may find application in the study of endometriosis. Chen et al. (2012) developed a microfluidic platform of endometriosis using endometrial stromal cells and peritoneal mesothelial cells and investigated peritoneal endometriosis pathologies [107]. Tantengco et al. (2022) developed a vagina–cervix–decidua platform and investigated the microbiota and inflammatory responses of the female genital tract [108]. Ahn et al. (2021) designed a vascularised endometrium-on-a-chip, representing the endometrial microenvironment, in vivo endometrial vascular angiogenesis, hormone response, and response to the contraceptive drug levonorgestrel, suggesting their model for the study of female reproductive diseases including endometriosis, cancer, and infertility [109].

Additionally, organoids have shown potential as investigative tools for endometriosis. Organoids are three-dimensional cellular complexes made by self-organising genetically stable stem cells from healthy or diseased tissue in vitro, representing the in vivo biological system [90]. The studies of Boretto et al. (2019) developed an organoid of eutopic and ectopic endometriosis which possessed the typical features of endometriosis, exhibiting long-term expansion in culture for stages I–IV [110]. The research of Esfandiari et al. (2021) applied organoids to study the role of epigenetics, specifically methylation alteration in endometriosis compared to that in tissue biopsy specimens [111]. Gnecco et al. (2023) developed an organoid coculture of human endometrial epithelial and stromal cells, modelling the menstrual cycle of healthy and diseased patients [112]. The research of Fitzgerald et al. (2019) developed self-renewing endometrial organoids which displayed ER expression and long-term storage capacity [113]. Endometriosis organoids possess the original in vivo characteristics and comprehensively represent the diversity of the disease, can be cultured long term, and are sources of omics information (genomics, proteomics) and hormonal response information [110]. Undoubtedly, microfluidics and organoids offer excellent experimental models for investigating the aetiology and response to therapy of endometriosis in a personalised manner. Regulatory frameworks and harmonised approaches are needed, however, to optimize such systems and to allow for reproducible studies corresponding to all types and stages of endometriosis compared to healthy endometrial tissue. Standardization will allow for the application of these cutting-edge innovative methods in clinically settings, aiming towards point-of-care diagnostics and disease management.

## 4. Advanced Therapeutic Medicinal Products (ATMPs) Towards Disease Treatment

Advanced therapy medicinal products (ATMPs) are novel medicinal products for the treatment of human disease and injury [114]. As a type of biologic, they are produced via biological expression systems such as those used to produce monoclonal antibodies (mAbs), interferons, novel vaccines modalities, etc. [115]. ATMPs include gene therapy, somatic cell therapy, and tissue-engineered products. With the current deficit in treatment options for endometriosis, novel approaches to alleviate symptoms or cure disease are required. ATMPs have found application in many disease states, including cardiovascular, Alzheimer’s, cancer, and rare diseases [116], where their application in endometriosis warrants investigation. The application of biologics based on immunotherapies has shown promise; however, there is a lack of clinical studies specific to endometriosis, as outlined elsewhere [117]. For example, bevacizumab, an anti-VEGF monoclonal antibody, may be used in combination with other therapies for the treatment of endometriosis-associated infertility and cancer [118]. The sample size is a major limitation to this study, however, as randomized control trials are needed to validate the results and to determine the influence of confounding variables.

### 4.1. CRISPR Technology and Endometriosis

Gene editing techniques may allow for the correction of the impaired genes involved in the development and progression of endometriosis. Identifying and correcting relevant gene mutations may allow for targeted treatment in patients cohorts. The use of clustered regularly interspaced short palindromic repeats (CRISPR) technology may find such application, as it has in the treatment of certain cancers, neurodegenerative diseases, and muscular dystrophy conditions [119]. CRISPR allows for the correction of errors or mutations in the genome and gene regulation in cells, which can prevent and cure disease states [120]. CRISPR technology, particularly the CRISPR/Cas9 system, is a rapid, cost-effective tool finding increasing applications in clinical and therapeutic areas, allowing for gene editing CRISPR systems to utilise vector and non-vector systems in order to insert the correct gene into the cell genome, replacing the incorrect gene sequence. Genetic polymorphism in oestrogen and progesterone receptors occur in the endometrial tissue [121], and CRISPR may allow for regulation of the ER, as decreasing its expression via genetic editing may reduce the progression of endometriosis lesions in vivo; similarly, adjusting the progesterone receptor may allow for improved response to contraceptive therapies. The studies of Wang et al. (2019) used a CRISPR/Cas9 system to knock out the ARID1A gene in a cell line to investigate progesterone resistance in endometrial cancer [122]; this system could also be applied to endometriosis studies. Identifying mutated or dysregulated genes and their associated pathways involved in the pathogenesis of endometriosis is essential in applying gene editing technologies for disease treatment. The studies of Balasubramanian et al. (2024) identified the germline heterozygous deletion of the mRNA editing enzyme subunit *APOBEC3B* in ca. 96% of endometriosis samples confirmed with detection in serum, endometrium, and healthy ovarian samples [123]. *APOBEC3B* germline deletion contributes to the pathogenesis of endometriosis via an increase in epithelial to mesenchymal transition, cellular proliferation, and inflammation markers and a decrease in apoptosis markers [123]. The human application of gene editing technologies is greatly hindered, however, by off-target effects, and CRIPSR technology is less associated with this issue than are other gene editing methods [119]. Additional obstacles include the heterogenicity of endometriosis between patients; the influence of multiple gene variants; epigenetic modifications; environmental factors, including exposure to endocrine disrupting chemicals (EDCs); individual variations in menarche and menstruation cycles; the presence of comorbidities; and patient lifestyle factors all impact disease progression [124]. Applying gene editing technologies for the diagnosis and treatment of endometriosis in a clinical setting is therefore hindered by the multifactorial nature of the disease. Currently, the application of gene editing technologies is not clinically feasible for endometriosis, but ongoing studies using many disease states highlight the potential of this novel therapeutic approach. Successful implementation would undoubtedly revolutionize the timely diagnosis and treatment of disease in a personalised manner.

### 4.2. Mesenchymal Stem Cell Therapy

Mesenchymal stem cells (MSCs) are multipotent stem cells which can be found in many tissues including bone marrow, placenta, adipose, and endometrial tissue and which can differentiate into cells of multiple lineages, e.g., chondrocytes, osteoblasts, adipocytes, and myoblasts [125]. This multipotent potential and immunomodulatory activity make them ideal candidates for cell-based therapies. Immunomodulation by MSCs occurs post-migration to the site of inflammation through interaction with the immune cells and the suppression of innate and adaptive immune responses [126]. The first MSCs-based therapy was developed for the immune modulation of graft-versus-host disease (GVHD), in which MSC infusion is well-tolerated in clinical applications [127]. MSCs secrete many bioactive molecules which may be involved in the regulation of the inflammation response, lymphocyte differentiation, and the promotion of the regeneration of injured tissues [37]. The immunosuppressive effects of MSCs on immune cell types, including macrophages and NK cells, promote their application in the treatment of inflammatory diseases [128]. Studies have demonstrated that MSCs therapy is related to the repair of homeostatic microenvironmental and endogenous stem cell function in patients following parenterally or systemically administered MSCs [129]. Preclinical and clinical studies have assessed the ability of MSCs to treat female infertility by improving the functions of the ovaries and uterus [130]. MSCs are known to contribute to endometrial regeneration and have been isolated from eutopic endometriosis implants [131]. Importantly, ectopic endometrial MSCs from patients with endometriosis demonstrated increased proliferation, migration, and angiogenic ability when compared the eutopic MSCs from the same patient [132]. Isolated MSCs for endometriosis lesions and healthy endometrium lack ER expression and as such, are not responsive to hormonal suppression therapy [132]. The studies of Abomaray et al. (2021) concluded that the use of MSCs as therapy for endometriosis will be hindered by their contribution to ectopic tissue growth [128]. Additionally, animal studies demonstrated a decreased expression of inflammatory IL-1β, IL-10, TNF, and IL-1R in endometriosis models post-MSC application [133]; importantly, the IL-6 and IL-8 expression levels increased, suggesting that a pro-inflammatory response also occurs.

Menstrual-blood stem cells are similar to endometrial stem cells and express excellent proliferation, differentiation, and regeneration abilities compared to those of bone marrow and adipose-derived stem cells [134]. Studies have successfully differentiated menstrual stem cells into endometrial cells using oestrogen and progesterone therapy in mouse models [135]. The stem cell theory of endometriosis, in which endometrial stem cells (and bone marrow stem cells) are involved in endometrial lesion formation, is supported by the fact that stem cells are also shed during menstruation [136]. Menstrual stem cells are easy to collect using non-invasive procedures in an ethical manner, with studies suggesting their application in the treatment of many diseases, including premature ovarian failure, liver failure, pulmonary issues, cardiac diseases, myocardial infarction and stroke, Alzheimer’s disease [137], and sciatic nerve injury, amongst others [38]. Menstrual stem cells also have immunomodulatory effects and can regulate cellular and humoral immune pathways [138]. Future research may establish the suitability of menstrual stem cells for the treatment of endometriosis. Currently, the results of MSCs therapy demonstrate their safety but provides limited information on their efficacy, as studies do not often move beyond phase 3 clinical trials [139]. Pharmacodynamic and pharmacokinetic factors influence application, and a lack of pre-clinical testing models, adherence to good manufacturing practices (GMP), and large-scale production limitations also impact their clinical application. Ethical issues also arise relating to donor consent, genetic modification, and a lack of universally accepted regulatory guidelines, which also impacts the clinical application of stem cell therapies [133]. The use of autologous or allogenic MSCs has its own benefits and limitations, and the allogenic approach is deemed more applicable [140]. The use of allogenic stem cells derived from the close relatives of the patient may reduce immune rejection and improve patient response. Much more specific research is needed to develop any such stem cell therapy, with pre-clinical and randomized control trials essential to determine clinical therapeutic use.

## 5. Future Direction Towards Patient Care

As awareness of endometriosis prevalence and its severe impact on patients becomes increasingly recognized, many studies regarding effective treatments and, if possible, prevention modalities, are warranted. As a multifaceted condition with numerous factors contributing to its aetiology, prevention protocols are unlikely to be established. Improved diagnostics, with accurate and timely intervention, will improve the quality of life of patients and improve fertility issues. Currently, the application of biomarkers for the diagnosis of endometriosis is not fully established or optimized; similarly, the potential treatment options discussed in this review are a long way from providing therapeutic benefits, as such techniques must first clear clinical trials. Current best approaches for treatment include therapeutics to limit disease progression, i.e., combined oral contraceptives, NSAIDs, gonadotropin-releasing hormone receptor antagonists and agonists, and progestins, often fail to alleviate symptoms, while exhibiting many side effects. Macrophage-based cell therapy as a novel therapeutic is emerging for many diseases [141], and it may offer benefits for endometriosis in the future. Autologous and allogenic macrophages derived from induced pluripotent cells, which can be loaded with additional medicinal agents, is a promising field of research [141]. The important research of Bendifallah et al. (2022) established an ENDO-miRNA methodology using a saliva-based diagnostic miRNA signature for endometriosis [142]. The researchers concluded that the method may offer a scalable diagnostic approach for endometriosis, at low-cost and available over all socioeconomic cohorts [142]. Future research directed at the large-scale application of such a method is required, as it offers benefits such as ease of sample collection, high selectivity, and high detectability of miRNA in saliva samples. At present, however, such modalities face the same limitations as those described in Section 4.2. In a clinical setting, information dissemination to patients regarding optimal diet programs and lifestyle choices may reduce symptoms and pain severity. As a heterogenic disease, individual approaches to management may result in improvement of symptoms. The removal of processed foods which contains EDC pesticides and pro-inflammatory chemicals, for example, with adherence to an anti-inflammatory organic diet is worth assessing. The impact of EDCs on hormone-based morbidities, including reproductive cancers and endometriosis, is an important consideration [124].

## 6. Conclusions

Endometriosis is a chronic debilitating disease, manifesting with an array of symptoms influenced by the stage and type present. Deep infiltrating endometriosis is considered the most severe form and is associated with infertility in afflicted women. It is well accepted that oestrogen plays a significant role in inducing inflammation, neuropathic pain, and neurogenesis. Oestrogen is associated with the release of IL-6 from macrophages, one of the main pro-inflammatory cytokines present in the endometriosis microenvironment. The use of oral contraceptives has been applied for the treatment of this disease as a first-line therapy. Many patients, however, are non-responsive to such approaches or are progesterone resistant. The role of oestrogen in endometriosis lesion progression is multifactorial; therefore, research is warranted to gain better insight into the relationship between oestrogen and pain, including nociceptive pain. Many patients undergo laparoscopic explorative and diagnostic surgery to accurately diagnose and excise endometriosis tissue. Relief is often short-term, however, with up to 50% of patients suffering lesion re-growth postoperatively; currently, there is no cure for this disease. As a comorbid condition associated with many diseases, including autoimmunity, cancer, and mental health issues, the diagnostic toolkit available to clinicals is limited, and misdiagnosis as IBS or IBD is a frequent occurrence. Diagnostic aids are urgently required to improve the time to diagnosis and response to treatment. Biomarkers, including immune mediators, e.g., cytokines, extracellular vesicles, microbial biomarkers, and novel approaches, such as organoids and microfluidics, may prove to be clinically useful. In conjunction with omics technology and machine learning, these assays provide enhanced sensitivity and specificity for the analysis of disease pathologies and treatment. Additionally, novel treatment approaches are warranted, in which biologics and ATMPs, such as MSCs and gene editing tools, may allow for treatment advances going forward. It is important to note, however, that the diagnostic modalities and treatment methods discussed here are still far from being clinically applicable, and their full potential and applicability require more intensive investigation. Until such time as clinical trials can be completed, these methods have no clinical application. The multifaceted aetiology and heterogenicity of the disease greatly hinders and restricts investigative research, with a lack of in vivo studies, pre-clinical animal models, and ethical issues limiting advances. The application of biologics such as immunotherapies has shown promise in the treatment of inflammatory disease; however, there is a lack of clinical studies specific to endometriosis. There is also a need for pre-clinical and clinical trials regarding the suitability of MSCs in the treatment of endometriosis, as such studies have investigated their efficacy in the treatment of female infertility. Without significant advances in the diagnostic and treatment modalities available, many women will continue to suffer chronically with poor quality of life, possible infertility, and associated mental health issues.

## Figures and Tables

**Table 1:** Stages of endometriosis and its scoring system, according to the revised American Society for Reproductive Medicine (rASRM) system [19].

Stage	Characteristics
I—minimal	Few superficial implants, mild adhesions. Score: 1 to 5 points
II—mild	Deeper implants and more adhesions than in stage I. Score: 6 to 15 points
III—moderate	Many deep endometrial implants, cysts in at least one of the ovaries; filmy adhesions may be present. Score: 16 to 40 points
IV—severe	A large number of cysts, severe adhesions, infertility. Score: >40 points

**Table 2:** Detailed symptomology, prevalence, and treatment considerations of the various types of endometrioses according to ENZIAN classification system.

Type	Area Affected	Symptoms	Prevalence	Treatment and Clinical Significance
Peritoneal or superficial endometriosis	Found mainly on the pelvic peritoneum	Dysmenorrhea, chronic pelvic pain, dyschezia	15–50% of endometriosis patients [33]	Pharmacological, involving oral contraceptives and analgesics and/or surgical treatment to remove ectopic tissue; 33% of patients are not responsive to progestin therapy [34], 20% of patients show no improvement post-surgery. Post-surgery recurrence rate of 30% to 50% [33], with some studies suggesting recurrence as high as 67% [9].
Ovarian endometriosis	Ovaries, fallopian tubes	Ovarian endometriomas/pseudocysts, or chocolate cysts (cystic lesions filled with dark endometrial fluid) [2], infertility, ovarian cancer [21]	17–44% of endometriosis patients [2]	
Deep infiltrating endometriosis	Uterosacral and cardinal ligaments, pouch of Douglas, posterior vaginal fornix, bowel and bladder [11]; tissue penetration > 5 mm, fibrosis and adhesions, extra-pelvic lesions [16]	Dyspareunia, dysmenorrhea, chronic pelvic pain, bladder and urinary symptoms, haematuria dyschezia, diarrhoea, constipation, intestinal cramping and bloating [4], infertility	20% of endometriosis patients [11]	

**Table 4:** Advantages and limitation of microfluidics in the study of endometriosis.

Advantages	Limitations
Can replicate the in vivo environment, with multiple cell types interacting.	May not allow for accurate hormonal alterations present in the female reproductive system [106].
Additional cells, such as immune cells, can be added for disease-specific studies.	Hormone heterogeneity among individuals with or without endometriosis.
Allows for therapeutic efficacy studies and personalised medicine.	Limited access to sources of renewable cells, i.e., primary cells.
Provides a highly controllable research environment [106].	Cell culture environment constraints.
Can be analysed using various techniques [104].	Limitations associated with the material used to produce the platforms [105].
Can assess metabolic processes, cytokine biomarkers, inflammation, and membrane degradation [13].	Currently, there is a lack of universal criteria for determining microfluidic platforms.
Standardization of systems is possible [105].	Difficult to grow endometrial tissue long-term.
